# Statistical analysis plan for the OPTIMUM study: optimising immunisation using mixed schedules, an adaptive randomised controlled trial of a mixed whole-cell/acellular pertussis vaccine schedule

**DOI:** 10.1186/s13063-021-05874-6

**Published:** 2022-02-07

**Authors:** James A. Totterdell, Gladymar Perez Chacon, Marie J. Estcourt, Mark Jones, Peter Richmond, Thomas L. Snelling, Julie A. Marsh

**Affiliations:** 1 School of Public Health, University of Sydney, Camperdown, Australia; 2 Wesfarmers Centre of Vaccines and Infectious Diseases, Telethon Kids Institute, Perth, Australia; 3 School of Population Health, Curtin University, Bentley, Australia

**Keywords:** Adaptive design, Statistical analysis plan, Randomised controlled trial, Pertussis vaccine, Food allergy

## Abstract

**Objective:**

The purpose of this double-blind, randomised, controlled trial is to compare allergic outcomes in children following vaccination with acellular pertussis (aP) antigen (standard of care in Australia) given at 2 months of age versus whole cell pertussis (wP) in the infant vaccine schedule.

**Participants:**

Up to 3000 Australian infants 6 to <12 weeks of age born ≥32 weeks gestation.

**Interventions:**

The intervention is a wP containing vaccine as the first scheduled pertussis vaccine dose instead of an aP containing vaccine.

**Outcomes:**

The primary outcome is a binary indicator of history of IgE-mediated food allergy at the age of 12 months confirmed, where necessary, with an oral food challenge before 18 months of age. Secondary outcomes include (1) history of parent-reported clinician-diagnosed new onset of atopic dermatitis by 6 or 12 months of age with a positive skin prick test to any allergen before 12 months of age, (2) geometric mean concentration in pertussis toxin-specific IgG before and 21 to 35 days after a booster dose of aP at 18 months of age, and (3) sensitisation to at least one allergen by 12 months of age.

**Results:**

Operating characteristics of trial decision rules were evaluated by trial simulation. The selected rules for success and futility approximately maintain type I error of 0.05 and achieved power 0.85 for a reduction in the primary outcome from 10% in the control group to 7% in the intervention group.

**Discussion:**

A detailed, prospective statistical analysis plan (SAP) is presented for this Bayesian adaptive design. The plan was written by the trial statistician and details the study design, pre-specified adaptive elements, decision thresholds, statistical methods, and the simulations used to evaluate the operating characteristics of the trial. Application of this SAP will minimise bias and supports transparent and reproducible research.

**Trial registration:**

Australia & New Zealand Clinical Trials Registry, ACTRN12617000065392. Registered on 12 January 2017

**Study protocol:**

10.1136/bmjopen-2020-042838

**Supplementary Information:**

The online version contains supplementary material available at (10.1186/s13063-021-05874-6).

## Introduction

Combination vaccines containing whole-cell pertussis (wP) antigens were phased out from the Australian National Immunisation Program (NIP) between 1997 and 1999 and replaced by vaccines containing less reactogenic acellular pertussis (aP) antigens. In a case-control study of Australian children born during the transition period, those with allergist diagnosed IgE-mediated food allergy were less likely to have received whole-cell vaccine in early infancy than matched population controls [odds ratio 0.77, 95% confidence interval (0.62, 0.95)] [1]. The notions of mechanistic plausibility that support these results are based on the Th_1_/Th_17_ immune-modulating properties of some components of *Bordetella pertussis* within wP formulations, which could promote the development of oral tolerance in children predisposed to maintain Th_2_- biased responses into later infancy [2].

We hypothesise that a single dose of whole-cell vaccine in early infancy is protective against IgE-mediated food allergy in early childhood.

This statistical analysis plan (SAP) provides a priori specification of the decision-making rules and the statistical methods to be used in a prospective clinical trial. The SAP was prepared after data collection had commenced, but prior to any unblinded data analyses. The coordinating principal investigator was responsible for approving the SAP, and it was reviewed and approved by the trial independent data monitoring safety board (DMSB). The SAP is written to be consistent with the CONSORT 2010 Statement and further guidelines and supports transparent and reproducible research.

## Study synopsis

The OPTIMUM trial is a two-armed, prospective, multi-site double-blinded, adaptive, randomised clinical trial designed to assess the effectiveness of a first scheduled dose of wP vaccine for preventing food allergy, compared to the standard aP vaccine in healthy, vaccine-eligible Australian infants aged 6 to <12 weeks born at ≥ 32 weeks gestation in Australia. The trial uses a Bayesian group-sequential design with pre-specified stopping rules informed by predictive probabilities of trial outcomes given the data available at interim analyses.

The protocol defines two stages of the trial. Stage 1 was designed to obtain detailed solicited adverse event data following each primary pertussis vaccine dose, and post-priming immune response data for the first 150 infants only. Stage 2 was designed as a simpler protocol with less intensive follow-up and fewer visits scheduled.

During stage one, 150 participants were enrolled at Perth Children’s Hospital (PCH), Western Australia, between March 2018 and January 2020. Stage 2 will expand the trial to include sites in Sydney and Melbourne randomising up to 3000 participants including the 150 from stage 1. The stage 1 and stage 2 schedules of assessment are available in the previously published protocol [2].

## Interventions

Eligible participants will be randomised to receive a 0.5-mL dose of the WHO-prequalified pentavalent formulation of diptheria-tetanus-pertussis (whole-cell)-hepatitis-B- *Haemophilus influenzae* type b vaccine (DTwP-HB-Hib) (PENTABIO®PT, Bio Frama, study vaccine), or else a hexavalent formulation of DTaP-HB-Hib plus inactivated poliovirus vaccine (IPV) (INFANRIX HEXA®, GlaxoSmithKline, comparator vaccine), as the first dose of the pertussis vaccination schedule between 6 and <12 weeks of age. This is given via intramuscular injection into the anterolateral aspect of the right thigh. Other scheduled 6-week doses are co-administered as per Australia’s National Immunisation Program.

## Study population

### Inclusion criteria

An eligible infant must fulfil all the following:

- Healthy male or female infant aged 6 to <12 weeks old.
- Born ≥32 weeks gestation.
- Parent or Legally Accepted Representative (LAR) who has the capacity to understand the parent information sheet and consent form (PISCF) and study related procedures.
- Parent or LAR is willing and able to give informed consent for participation in the trial.
- Infant known to be free of significant medical problems as determined by a medical history and clinical examination by a medically qualified investigator.
- Parent or LAR has access to a telephone.
- Parent or LAR who is able and willing to comply with the requirements of the protocol in the opinion of an investigator.
- Parent or LAR is willing to allow other parties involved in the treatment of their child (including general practitioner, medical centre staff, and any other medical professionals the child may be a patient of for the duration of the trial) to be notified of their participation in the trial and for participation in the trial to be recorded within the Australian Immunisation Register (AIR). The parent/LAR is willing to allow the study team to obtain a vaccination history from AIR and/or local provider.
- Parent or LAR is willing to allow the study team to obtain information from the infant’s doctor, other health care professionals, hospitals, or laboratories concerning the infant’s health from enrolment until 1 month after the 18-month vaccinations.
- Infant is available for the entire study period.

### Exclusion criteria

The participant may not enter the trial if any of the following apply:

- History of pre-existing parent-reported clinician diagnosed IgE-mediated food allergy.
- History of parent-reported, clinician-diagnosed pertussis infection.
- Receipt of any prior vaccine, except for a single birth dose of hepatitis B vaccine within the first 7 days of life (only for visit 1).
- Contraindication to any routine infant immunisation: history of allergy, including anaphylaxis, to any vaccine or vaccine component.
- Contraindication to paracetamol.
- Receipt of investigational vaccines/drugs, other than the vaccines used in the study, since birth or their planned use during the study period, until the final study visit (i.e. at approximately 19 months of age).
- Receipt, or planned receipt, of any non-routine vaccines within 14 days after the first dose of pertussis containing vaccine.
- Receipt of more than 2 weeks of immunosuppressants or immune modifying drugs (e.g. prednisolone >0.5 mg/kg/day).
- Serious chronic illness including severe congenital anomalies affecting heart, brain, and/or lungs.
- History of any neurologic disorders or seizures.
- Administration of immunoglobulins and/or any blood products since birth or planned administration during the study period.
- Planned travel to any region that remains at risk of a poliomyelitis transmission at any time before study visit 8 for stage 1, and for stage 2, before the final phone/electronic contact at approximately 19 months of age.
- Parents or LAR who plan to move out of the geographical area where the study would be conducted.
- Any other significant disease or disorder which, in the opinion of the investigator, either may put the participants at risk because of participation in the trial, or may influence the result of the trial, or the participant’s ability to participate in the trial.

### Temporary exclusion criteria for vaccination

- Fever (≥38 ^∘^C as determined by axillary assessment) and/or acute disease at the time of recruitment or at any study visit where vaccination will occur as defined by the presence of a moderate or severe illness with or without fever (with the exception of minor illnesses such as diarrhoea, mild upper respiratory infection without fever). In such situations, randomisation or the study visit should be postponed until the participant has recovered.
- For stage 1 (visits 2, 3, and 7 only), receipt of any vaccination with a licensed vaccine product, including seasonal influenza vaccination or meningococcal vaccination, within the preceding 14 days. In these situations, the study visit should be deferred until 14 days have elapsed.

### Exclusion from per-protocol population

The following criteria will be checked at each visit subsequent to the first visit. If any become applicable during the study, it will not require automatic withdrawal of the participants from the study, but may determine the participant’s evaluability in the per-protocol (PP) analysis. The data would, however, continue to be included in the intention-to-treat (ITT) analysis.

- Use, or planned use, of any investigational or non-registered product (drug or vaccine) other than the study vaccine(s) during the study period, until 1 month after the final study visit (i.e. at approximately 20 months of age).
- For stage 1 only: Chronic administration (defined as more than 14 days) of immunosuppressants or other immune-modifying drugs during the study period (for corticosteroids, this will mean prednisone ≥0.5 mg/kg/day, or equivalent. Inhaled and topical steroids are allowed).
- For stage 1 only:
  - Administration of a vaccine not foreseen by the study protocol within 14 days after any scheduled vaccine dose.
  - Administration of immunoglobulins and/or any blood products during the study period.
  - Administration of any of the vaccines used in the study outside of the stipulated period.
  - Administration of any live vaccines, those containing tetanus- or diphtheria-related antigens or those that contain bacterial lipo-polysaccharide or other adjuvanted vaccines between study visit 3 and study visit 5.

## Objectives and outcomes

### Primary

The primary objective is to determine whether, compared to the standard strategy of 3 priming doses of aP (aP-only), a mixed wP/aP schedule (first dose of wP followed by doses of aP) protects against the development of IgE-mediated food allergy.

The primary outcome is history of IgE-mediated food allergy at the age of 12 months and confirmed, where necessary, by oral food challenge (OFC) before 18 months. A participant will be considered to have the outcome if there is evidence of sensitisation to a food on skin prick test (SPT), and either:

- Unequivocal IgE-mediated food allergy, defined as (i) a positive oral food challenge, or (ii) history of clinician-diagnosed food anaphylaxis, with symptoms affecting at least 2 of the following: skin, gastrointestinal tract, respiratory tract, and cardiovascular system,
- Highly probable IgE-mediated food allergy, defined as history of food allergic reaction consistent with PRACTALL criteria [3].

### Secondary

The protocol defines a number of secondary objectives and associated outcomes. These are numbered below.

#### Clinical and mechanistic

1. Compare the rate of new onset atopic dermatitis in each study group: two binary outcomes will be investigated:
  - A history of parent-reported clinician-diagnosed new onset atopic dermatitis by 6 months of age and a positive SPT to any allergen by approximately 12 months of age
  - A history of parent-reported clinician-diagnosed new onset atopic dermatitis by 12 months of age and a positive SPT to any allergen by approximately 12 months of age.
2. Compare the rate of SPT positivity to common allergens in each study group: two binary outcomes will be investigated:
  - A SPT wheals >1mm greater than the negative control to at least one allergen by approximately 12 months of age
  - A SPT wheals ≥3mm greater than the negative control to at least one allergen by approximately 12 months of age.

#### Stage 1: Atopic immunophenotypic response and vaccine response

3 Compare the Th2 immunophenotypic response to tetanus toxoid and egg antigens in infants in each study group: IgE concentrations to the following antigens will be collected at approximately ages 6 (immediately prior to third pertussis-containing vaccine dose) and 7 months (21-35 days after vaccination) using ImmunoCAP total and antigen specific IgE assays (Thermo Fisher Scientific/Phadia, Uppsala, Sweden) during stage 1 only (150 participants):
  - Total IgE concentration
  - IgE concentration to tetanus toxoid vaccine antigen
  - IgE concentration to egg white antigen
  - IgE concentration to whole egg antigen.
  Assays will be in kU/L per sample. IgE levels ≥0.10 kU/L are considered positive. The total IgE low-range assay can detect concentrations between 0.1 and 100 kU/L. The total IgE high-range assay has range 2 to 5000 kU/L. The specific IgE are reported in the range 0.00 to 100.00 kU/L with results above 100.00 kU/L reported as above this limit.

4 Compare vaccine antibody responses in each study group: vaccine antigen-specific IgG concentrations/titres will be measured at approximately ages 6, 7, 18, and 19 months using a multiplex fluorescent bead assay [4, 5], for the following during stage 1 only (150 participants).
  - Pertussis toxin (PT)
  - Filamentous haemagglutinin (FHA)
  - Pertactin (PRN)
  - Tetanus toxoid (TT)
  - Polyribosylribitol phosphate (Hib-PRP)
  - 13-valent pneumococcal vaccine (serotypes 1, 3, 4, 5, 6A, 6B, 7F, 9V, 14, 18C, 19A, 19F, and 23F)
  - Hepatitis B surface antigen (HBsAg)
  - Diphtheria toxoid (DT)
  The seropositive thresholds for pertussis antigens are:
  ≥5 IU/mL for PT, FHA, and PRN
  The seroprotective thresholds other antigens are:
  ≥0.1 IU/mL for TT [6] and DT
  ≥10 mIU/mL for HBsAg
  ≥1*μ*g/mL for Hib-PRP [7]
  ≥0.35*μ*g/mL for pneumococcal serotypes [8, 9]
  For each antigen, we will assess the ratio of the geometric mean titres (GMT) or concentrations (GMC) across study groups at each time point, the fold-rise in concentration from 18 (immediately before boosting with a dose of DTaP with inactivated polio vaccine) to 19 months (21-35 days after vaccination), and proportions of seroprotection or seropositivity. A 4-fold rise or greater will be considered indicative of seroconversion. For pneumococcal IgG responses, the lower limit of quantification ranges from 0.3 to 1.9 ng/mL [10]. For the other antigen-specific IgG, the lower limits of quantification range from 0.01 mIU/mL (DT) to 0.43 mIU/mL (FHA) [5].

#### Stage 2: Vaccine response

5 Compare vaccine antibody responses in each study group: vaccine antigen-specific IgG concentrations (PT, FHA, PRN, TT, Hib-PRP, 13-valent pneumococcal, HBsAg, DT) as previously listed for stage (1) will continue to be measured at ages 18 and 19 months in stage 2 up to a maximum of 300 participants enrolled at PCH/TKI unless non-inferiority of wP/aP compared to aP is established earlier.

### Safety and tolerability

To assess the local and systemic adverse reactions (ARs) experienced by participants in each study group after administration of the pertussis containing vaccine doses from the day of vaccination (day 0) until 7 days later (day 6), solicited adverse events and intensity grading will be collected by diary cards for the 7 days after vaccination (for participants in stage 1 vaccinations at 2, 4, 6, and 18 months of age and the first 300 participants in stage 2 enrolled at TKI for vaccinations at 2 and 18 months of age). The following solicited adverse events (AEs) will be collected:

- Axillary temperature (fever ≥38 ^∘^C)
- Irritability
- Restlessness
- Vomiting
- Diarrhoea
- Anorexia
- Drowsiness
- Injection site reactions (redness, swelling, induration, and pain).

The definition and intensity grading for fever, diarrhoea, and local reactions (except for immunisation site pain) follows the Brighton Collaboration guidelines [11–15]. For other solicited adverse events, intensity grading scales based on impact on daily activities are used (grade 0 or absent; grade 1 if easily tolerated; grade 2, sufficiently discomforting to interfere with normal everyday activities; and grade 3, if prevents normal everyday activities or requires significant medical intervention).

### Vaccine satisfaction

To assess parental dissatisfaction with vaccination in each group after each scheduled vaccine encounter, the following will be collected:

1. Stage 1 infants—Primary caregiver reported satisfaction with the vaccination experience on a 5-point Likert scale (from very satisfactory to very unsatisfactory) on day 7 after vaccination at 2, 4, 6, and 18 months of age
2. Stage 2 infants, first 300 enrolments at TKI—Primary caregiver reported satisfaction with the vaccination experience on a 5-point Likert scale (from very satisfactory to very unsatisfactory) on day 7 after vaccination at 2 and 18 months of age.

## Randomisation

Eligible participants will be randomised in 1:1 allocation to receive the study or comparator vaccine. Randomisation is by computer-generated allocation sequence prepared by the trial statistician [JT] and based on randomly permuted blocks of size 6, 8, or 10, and stratified by study site. The randomisation codes are password-protected and held by the trial statistician.

## Blinding

An electronic copy of the randomisation list is distributed by the study statistician to the unblinded pharmacist for printing and concealment. The randomisation list is concealed from all blinded research staff in a non-transparent envelope until completion of the study. An unblinded pharmacist or research nurse obtains the next contiguous allocation from the concealed list (i.e. the lowest available randomisation number) and prepares the study or comparator vaccine into a clear 1-ml ready-to-administer syringe, labelled with the study participant’s number and their identifiers. The syringes are covered to prevent unblinding. At enrolment, vaccines may be administered by either a blinded, or an unblinded nurse. If unblinded, this nurse has no further involvement in the follow-up of the participant. Parents and all other research staff remain blinded until study completion.

To maintain blinding while ensuring all participants receive at least three priming doses of IPV, a dose of DTaP-IPV (INFANRIX®IPV, GlaxoSmithKline, catch-up vaccine), in lieu of DTaP, is administered to all participants at age 18 months.

The blinding process may be broken under compelling medical or safety circumstances. Code breaks will be authorised by the coordinating principal investigator and will be communicated directly to the parents and/or medical team by the trial statistician.

## Sample size

A maximum sample size of 3000 participants is planned; 150 participants in stage 1 and up to 2850 in stage 2. In accordance with trial simulations of the above design, we estimated the trial would have 85% power to detect a reduction in IgE-mediated food allergy by 12 months of age from 10 to 7% while controlling the probability of a type I error at approximately 5%. This effect size was selected on the basis of consultation with clinical stakeholders.

The sample size for this study is adaptive; therefore, the actual trial sample size may be less than 3000 participants. Based on simulations undertaken for the trial design, the actual sample size is likely to be at least 1000 participants given the expected accrual rates and timing of the first interim analysis. We estimated a 69% probability of stopping early for futility if the null were true, and in the alternative scenario (reduction from 10 to 7% in the primary outcome), we estimated 59% probability of stopping early for expected success.

## Statistical analysis

### Baseline characteristics

All subjects who were invited to participate in this trial will be accounted for, and a CONSORT flow chart will be prepared for both the stage 1 and stage 2 participants. Reasons for early withdrawal will be listed for all participants who prematurely withdraw from the study. The number of participants who are screened but not randomised will be presented and the reasons for their non-participation will be listed (where available). The number of participants who are randomised, the number who fulfil eligibility criteria, and the number who satisfy elimination criteria at the specified time-points in the study will be summarised. The number of participants randomised by study centre and the rate of accrual over time will also be reported.

The trial participants will be summarised according to baseline variables. The data for each baseline variable will be presented descriptively. Quantitative variables will be summarised by median and inter-quartile range (IQR). Qualitative variables will be summarised by counts and proportions.

Summaries of the following listed baseline data will be reported.

- Demographics and infant’s medical history:
  - Site of enrolment
  - Age at enrolment (days)
  - Sex (male/female)
  - Place of residence (state)
  - Number of biological siblings and birth order (integer)
  - Infant and parental ethnicity
  - Parental country of birth
  - Parental education (highest achievement: primary school, secondary school, TAFE or trade certificate, bachelor-level university degree, post-graduate university qualification)
  - Combined parental income (≤ $18,000, $18,001 to $37,000, $37,001 to $87,000, $87,001 to $180,000, > $180,000)
  - Child attends daycare (yes/no, days per week)
  - Pet ownership (dog or cat at home; kept inside, outside, or both)
  - Breastfeeding status (exclusively, partially, or not fed with breast milk in the week before enrolment)
- Birth history:
  - Maternal gravidity and parity (integers)
  - Delivery type (vaginal, forceps/vacuum assisted, elective Cesarean section, emergency Cesarean section)
  - Intrapartum antibiotics (yes/no)
  - Neonatal systemic antibiotics (yes/no)
  - Gestational age at delivery (weeks and days)
  - Birth weight (grams)
  - Length (cm)
  - Head circumference (cm)
  - Apgar score at 1 and 5 min (0 to 10)
  - Hepatitis B vaccine at birth (yes/no)
- Maternal immunisation history confirmed where possible on Australian Immunisation Register (AIR) or through vaccination providers:
  - Maternal receipt of pertussis booster vaccination in the preceding pregnancy (yes/no) and type of pertussis-containing vaccine given
  - Maternal receipt of pertussis booster vaccination in the preceding 5 years (not including preceding pregnancy) (yes/no)
  - Maternal seasonal influenza vaccination during preceding pregnancy (yes/no)
- Family history of atopic diseases:
  - At least one first degree relatives with any clinician diagnosed atopic disease (asthma, allergic rhinitis, atopic dermatitis, IgE-mediated food allergy, no family history of atopy)
- Physical examination at enrolment:
  - Temperature (Celsius scale)
  - Weight (grammes)
  - Length (cm)
  - Head circumference (cm)

### Analysis sets

The planned analysis populations are defined as in Table 1.

**Table 1:** Pre-defined analysis sets to be used in summaries and analyses

Population	Description
Intention-to-treat (ITT)	All randomised participants with their treatment group as randomised. Participants who received vaccines/medications violating the exclusion criteria but were enrolled will be included.
Per-protocol (PP)	All randomised participants who received as planned the vaccine strategy to which they were randomised, and who completed the whole study period according to the protocol (satisfied inclusion and exclusion criteria, and visits as scheduled).
Stage 1 assays (ITT)	All participants in the ITT set who were enrolled under stage 1 protocol at the Perth Children’s Hospital (PCH)/Telethon Kids Institute (TKI).
Stage 1 assays (PP)	All participants in the per-protocol set who were enrolled under stage 1 protocol at PCH/TKI site and who met none of the stage 1 per-protocol exclusion criteria and who did not receive any contraindicated vaccines or medications for stage 1.
Stage 1+2 assays (ITT)	All participants in the ITT who were enrolled under stage 1 protocol or under the stage 2 protocol at PCH/TKI who consented for the blood samples.
Stage 1+2 assays (PP)	All participants in the stage 1 assay set with the inclusion of the additional participants enrolled under the stage 2 protocol at PCH/TKI who consented for the blood samples and who satisfied all stage 1 and 2 inclusion/exclusion criteria.

### Analysis of primary outcome

Descriptive counts and proportions of the primary outcome will be tabulated by treatment group, including the number of inconclusive results.

Inferences for the primary outcome will be based on Bayesian models. Each model will be inferred separately for the ITT and PP analysis sets. Interest lies in the measure of effect of assignment to the study vaccine on the primary endpoint relative to the comparator vaccine, denoted by *δ*. Depending on the model, this might represent the difference in proportions meeting the primary outcome, or some transformation of these proportions (log odds-ratio). In either case, the primary statistical hypotheses are:

$$\mathrm{H}_{0}:\delta\geq 0\ \text{vs.} \mathrm{H}_{1}:\delta <0 $$

At the final analysis, evidence of effect will be quantified as the posterior probability that *δ*<0 (that is, assignment to the study vaccine reduces the risk of IgE-mediated food allergy), conditional on the specified model given the data observed. If this posterior probability meets a pre-specified threshold of evidence, *q*, then a decision of superiority is recommended at the final analysis (trial success). The decision rule is (conditional on data *D*):

$$d(D) = \left\{\begin{array}{ll} \text{success} & \text{if}\ \mathbb{P}(\delta<0|D)>q \\ \text{failure}\ & \text{otherwise}. \end{array}\right. $$

For this study, the threshold for assessing superiority is *q*=0.95. This value was chosen based on simulation studies investigating the effect of the interim analysis stopping rules on the probability of false positives, false negatives, and expected sample size.

Although a conclusion of superiority is recommended by the formal test of the above hypotheses, effect sizes will be presented as summaries (mean, median, and 95% credible interval (CrI)) of the posterior density obtained at the final analysis in addition to the posterior probability of superiority.

The primary analysis of the outcome will use independent Beta-Binomial models for the response proportion in the two study arms. In this case, *δ*=*θ*_*w*_−*θ*_*a*_, the difference in probability of IgE-mediated food allergy between the two vaccine strategies. In this analysis, data will be pooled across sites. Beta(1,1) priors will be assumed for both response probabilities.

### Adjusted analysis

A secondary supportive analysis of the primary outcome will use logistic regression to estimate a covariate adjusted odds ratio associated with the study vaccine strategy. The prognostic baseline covariates to be included as fixed effects are:

- Sex
- Study site
- Breastfeeding status (exclusively, partially, or not fed with breast milk in the week before enrolment)
- Birth order (first born versus subsequent birth order)
- Combined parental income (≤ $18,000, $18,001 to $37,000, $37,001 to $87,000, $87,001 to $180,000, > $180,000)
- Mode of delivery (Cesarean section versus not Cesarean section)
- Family history of atopic disease (first degree relative with food allergy versus not)

We will specify weakly informative Student *t*priors for regression coefficients with 4 degrees of freedom, 0 mean, and standard deviation of 1.75. Inference will be based on Markov chain Monte Carlo (MCMC) samples.

### Interim analyses

This trial includes interim analyses to be undertaken at pre-specified sample sizes to recommend whether the trial should be stopped early for futility or expected success.

The recommendations are made in accordance with pre-specified stopping rules based on the data available at the time of the interim. The decisions on whether to stop are informed by Bayesian predictive probabilities of treatment effect [16].

Interim analyses begin after 200 subjects have complete primary outcome data. Subsequent interim analyses will occur after every additional 200 subjects with complete primary outcome data until either a stopping rule is met or enrolment completed. Once a decision to stop has been made, or enrolment has reached the maximum sample size, enrolment will cease and all participants will be followed to study completion.

Due to the timing of the primary outcome, the number of interim analyses is conditional on enrolment rates. Under the assumed accrual rate (Table 2), approximately 10 interim analyses are expected.

**Table 2:** Summary of trial scenarios explored

Parameter	Values considered
Accrual	Constant rate of 16 per week (required accrual)
	Constant rate of 5 per week (slow accrual)
	Ramp-up (slow initially, increasing over course of study)
Time to outcome	Uniform between 48 and 72 weeks
Control response	0.1
Treatment response	0.05, 0.06, 0.07, 0.08, 0.09, 0.10
Superiority threshold	0.95, 0.955, 0.96, 0.965, 0.97
Success threshold	1, 0.975, 0.95, 0.925, 0.9, 0.8
Futility threshold	0, 0.025, 0.05, 0.075, 0.1, 0.2

The process for interim decisions is as follows. Let $\mathbb P(\delta < 0|D_{k})$ denote the posterior probability of superiority under the specified model conditional on data, *D*_*k*_, at interim *k*=1,...,*K*. We specify a prediction model, $f(\tilde D_{k}|D_{k})$ for future outcome data $\tilde D_{k}$ that uses the data observed so far, marginalised over any uncertainty in the prediction model parameters. Using predictions of the primary outcome for subjects enrolled who are yet to complete follow-up, as well as subjects yet to be enrolled, the stopping decision is based on the predicted outcome of the trial (in terms of the decision rule used at the final analysis) if enrolment were stopped now (with follow-up completed on enrolled participants), or continued to the maximum sample size, given what has been observed up to interim *k*.

The predicted probability of success (PPoS) [17] is calculated as

$$\text{PPoS}_{k}(q) = \mathbb E_{\tilde D_{k} | D_{k}}\left[\mathbf{1}_{(q,1]}\left\{\mathbb P(\delta<0|D_{k},\tilde D_{k})\right\}\right] $$

and is the probability that superiority would be decided at a final analysis using threshold *q* and future data $\tilde D_{k}$ according to $f(\tilde D_{k}|D_{k})$.

The value of PPoS_*k*_ depends on the specified prediction model. In this trial, the posterior predictive distribution of the primary analysis model is used as the prediction model. This is two independent Beta-Binomial distributions conditional on the observed responses in each treatment group.

#### Stopping rules

Two stopping rules are specified: (1) expected success and (2) futility. At each planned interim analysis *k*=1,2,...,*K*, let $n_{i}^{k}$ denote the number of participants in arm *i* who have reached the primary endpoint by the time of interim *k*. Let $y_{i}^{k}$ denote the number of participants in arm *i* out of $n_{i}^{k}$ who met the primary outcome criteria, and *D*_*k*_ denote the available data. In addition to the participants with observed outcomes at interim *k*, there are $m_{i}^{k}$ participants enrolled in arm *i*, of which some unknown $\tilde w_{i}^{k}$ will have a future response. There are also $l_{i}^{k}$ participants who could still be enrolled into arm *i* (in 1:1 allocation up to maximum sample size) of which some unknown $\tilde z_{i}^{k}$ will have a future response.

Using the prediction model and primary analysis model, the following values are calculated

$$\begin{aligned} \text{PPoS}_{k}^{0}(q) &= \mathbb E_{\tilde w_{1}^{k},\tilde w_{2}^{k}|y_{1}^{k},y_{2}^{k}}\left[\mathbf{1}_{(q,1]}\left\{\mathbb P\left(\delta<0|y_{1}^{k},y_{2}^{k},\tilde w_{1}^{k},\tilde w_{2}^{k}\right)\right\}\right] \\ \text{PPoS}_{k}^{1}(q) &= \mathbb E_{\tilde w_{1}^{k},\tilde w_{2}^{k},\tilde z_{1}^{k},\tilde z_{2}^{k}|y_{1}^{k},y_{2}^{k}}\left[\!\mathbf{1}_{(q,1]}\left\{\mathbb P\left(\delta<0|y_{1}^{k},y_{2}^{k},\tilde w_{1}^{k},\tilde w_{2}^{k},\tilde z_{1}^{k},\tilde z_{2}^{k}\right)\right\}\right]. \end{aligned} $$

The decision rule used for recommendations at interim analyses (assuming at least some number of participants are still to be enrolled to achieve the maximum sample size) is

$${}d(D_{k})=\left\{\begin{array}{ll} \text{stop for expected success} & \text{if}\ \text{PPoS}_{k}^{0}(q) > \overline{c}_{k} \\ \text{stop for futility} & \text{if}\ \text{PPoS}_{k}^{1}(q) < \underline{c}_{k} \\ \text{continue to analysis }k+1 &\text{otherwise} \end{array}\right. \quad k=1,...,K $$

where $q=0.95, \overline {c}_{k}=0.95$, and $\underline {c}_{k}=0.05$ for all *k* which were selected based on simulations (see Operating characteristics).

### Analysis of secondary outcomes

#### Clinical and mechanistic

Based on the intention-to-treat (ITT) and per-protocol (PP) analysis sets, the proportion of participants with each clinical and mechanistic outcome response will be reported. Unadjusted posterior summaries for the difference in proportions between the study vaccine and comparator will be calculated using beta-binomial models as for the primary outcome. Adjusted analyses will use logistic regression models where posterior summaries of the adjusted odds ratio associated with receiving the study vaccine will be reported along with any other model parameters. These models will adjust for the same covariates as in the adjusted model for the primary outcome and use the same priors.

#### Laboratory—stage 1 atopic immunophenotypic response

The sensitivity of the assay for total IgE and antigen-specific IgE concentrations will be reported. Results outside the limits of detection will be reported as below or above the limit of quantification. For analyses of concentrations, measurements will be log_10_ transformed from the unit scale and concentrations outside detection limits will be treated as censored.

For each outcome, we will report by treatment group and visit for the stage 1 assay analysis sets the following descriptive summaries:

- The median, IQR, and range of concentrations
- The proportion of missing concentrations
- The proportion of concentrations below or above limits of quantification
- The proportion of concentrations exceeding the positive response threshold (see page 38)
- Kaplan-Meier plots of concentrations

Inferences for geometric mean concentrations (GMC) and geometric mean ratios (GMR) between study groups will assume censored normal regression models for log-concentrations. Univariate models will be fit for each visit with treatment-specific means, and a multivariate model for all visits with unstructured covariance matrices and visit by treatment-specific means. If issues arise with the covariance structure, a simpler covariance model will be assumed, such as heterogeneous compound symmetry. Priors on mean parameters will be normal with mean 0 and standard deviation 10. An LKJ(1) prior is specified on the correlation matrix, and half-Cauchy with location 0 and scale 10 on the marginal standard deviation parameters. If the assumptions of the censored regression model are inappropriate then two-part models may be considered to separate modelling of detection and concentration at each visit.

Inferences for relative proportions exceeding positive response threshold by study group will assume logistic regression models with a fixed term for treatment group, visit, and their interaction, and random intercepts for participants.

#### Laboratory—stage 1 and 2 vaccine response

The sensitivity of each assay will be reported. Results outside the limits of detection will be reported as below or above the limit of quantification. For analyses of concentrations, measurements will be log_10_ transformed and concentrations outside the detection limits will be treated as censored.

For each outcome, we will report by treatment group and visit for the stage 1 and stage 1+2 (PT only) assay analysis sets:

- The median, IQR, and range of concentrations
- The proportion of missing concentrations
- The proportion of concentrations below the limit of quantification
- The proportion of concentrations exceeding the seroprotective/seropositivity thresholds (see page 38)
- The proportion of participants with a 4-fold or greater increase in their concentration from 18 to 19 months
- Kaplan-Meier plots of concentrations

Inferences for GMC, GMR, and proportions will use the same modelling approach as for the atopic immunophenotypic response outcomes.

Non-inferiority of the wP vaccine strategy compared to the standard aP vaccine strategy with respect to PT will be assessed against a non-inferiority margin of 2/3 on the GMR [18]. That is, if the GMR for a particular antigen and visit is defined as

$$\text{GMR} = \frac{\text{GMC}_{\text{wP}}}{\text{GMC}_{\text{aP}}} $$

then non-inferiority will be evaluated according to

$$\mathbb P\left(\text{GMR}\geq \frac{2}{3}\Big\vert \text{data}\right). $$

### Safety and tolerability outcomes

The number and proportions of infants in each study group with each type of solicited AE and their intensity grading will be summarised for each day following vaccination (day 0 to 6) for each vaccine occasion (2, 4, 6, and 18 months in stage 1; 2 and 18 months in stage 2). The number and proportion of infants in each study group with each type of solicited AE, and at least one type of solicited AE for each vaccine occasion will be reported.

### Vaccine satisfaction

The responses for vaccination satisfaction of primary caregivers for infants in each study group on the 5-point Likert scale on day 7 after each vaccine occasion will be summarised by counts and proportions. The number of vaccination experiences reported as either unsatisfactory or very unsatisfactory on the Likert scale will be reported by study group as counts and proportions.

### Subgroup analyses

No pre-specified subgroup analyses will be undertaken; however, some subgroup effects may be investigated as part of post hoc analyses. Any such subgroup analyses will be noted as being post hoc.

### Missing data

Reasons for missingness will be summarised. Patterns and predictors of missingness will also be investigated and summarised. Descriptive and summary statistics will generally be based on complete-cases data. The primary analyses will be based on complete-cases. Secondary analyses using multiple imputation for missing responses and covariates will be undertaken to investigate sensitivity of the results.

### Sensitivity analyses

Sensitivity of results to the specified priors will be investigated by estimating parameters under different prior configurations than those used in the primary analyses. In particular, the hyperparameter for prior variances will be varied to assess how conclusions may change under varying strength of the prior.

### Software

All data cleaning, summaries, and exploratory and pre-specified analyses will be undertaken using R [19]. For models where MCMC sampling is required, we will use Stan [20].

### Reporting

Results for the stage 1 atopic immunophenotypic response, vaccine response, and safety and tolerability outcomes will be reported by study group once complete follow-up has been obtained for the 150 participants in stage 1 up to their 7-month visit. This will not include SAEs or unsolicited AEs.

## Operating characteristics

The operating characteristics of the OPTIMUM trial were estimated using Monte Carlo methods assuming various scenarios for the data generating process.

**Fig. 1:**
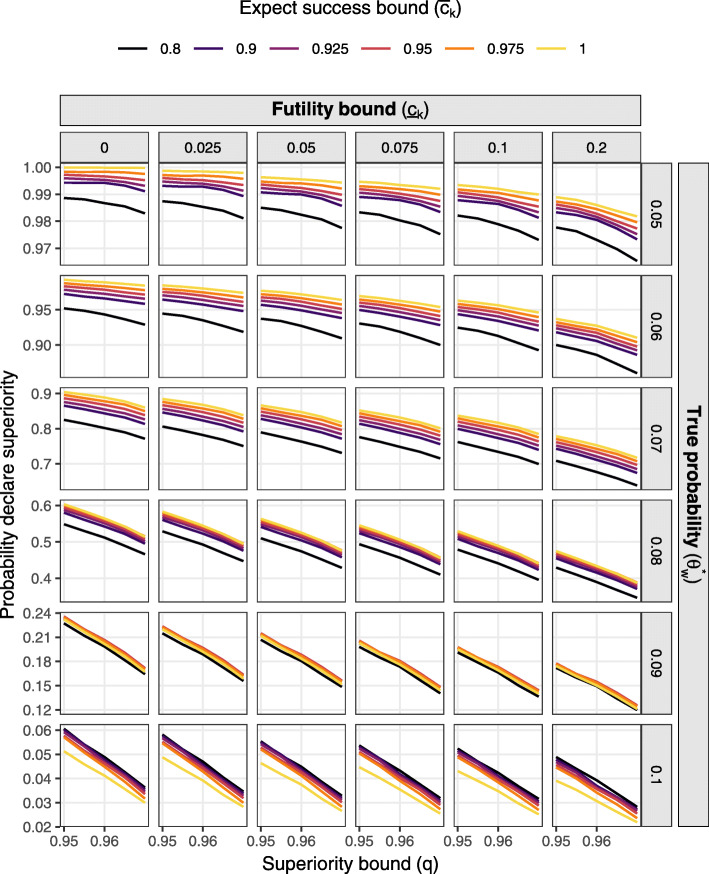
Probability of deciding superiority at the final analysis by decision thresholds and effect size assuming constant 16 per week accrual

The final thresholds were selected informally on the basis of their type I error, type II error, and expected sample size. For example, based on the final row of Fig. 1, tresholds consistent with type I error of approximately 0.05 were identified. These threshold combinations were then assessed for power and expected sample size, noting that many combination options result in similar opearting characteristics. Due to the common use of 0.95 as a one-sided threshold, the value *q*=0.95 was selected.

### Scenarios

A first interim analysis is scheduled to occur when primary endpoint data is available on 200 subjects. Subsequent interim analyses occur after every additional 200 subjects with available endpoint data until a stopping rule is met, or enrolment is already complete at the maximum sample size of 3,000.

Trial data was generated allowing variations in accrual rate and pattern of accrual, time to primary endpoint, true response probability in the control and treatment arms, and decision thresholds (Table 2).

Accrual was assumed to follow a non-homogeneous Poisson process and we investigated varying the baseline rate and varying the shape of accrual by allowing for slow initial accrual and a ramp-up in the latter stages of the trial. We assumed baseline accrual of 16 per week, and 5 per week (slow accrual is worst case for false positives), and a gradually increasing rate of accrual.

Time to primary outcome was assumed to be uniform between 48 and 72 weeks (mean 60 weeks) post-randomisation.

The probability of food-allergy within the standard aP vaccine strategy, $\theta _{a}^{\star }$, and study wP vaccine strategy, $\theta _{w}^{\star }$, populations investigated were $\theta _{a}^{\star }=0.1$ and $\theta _{w}^{\star }\in \{0.05, 0.06, 0.07, 0.08, 0.09,0.1\}$. Data were generated as Bernoulli random variables using the specified probability with time of enrolment generated from the accrual specification, and time to response generated from the time to outcome specification.

A grid of decision threshold values was investigated across all scenarios. For the final analysis, posterior probability thresholds of *q*∈{0.95,0.955,0.96,0.965,0.97} were investigated. Futility thresholds were fixed across all interim analyses and set to $\underline {c}\in \{0,0.025,0.05,0.075,0.1, 0.2\}$. Expected success thresholds were fixed across all interim analyses and set to $\overline {c}\in \{0.8,0.9,0.925,0.95,0.975,1\}$.

### Results

Simulation results from all scenario combinations were based on 10,000 replications performed using R [19]. Results are presented here only for the 16 per week accrual scenario; the other scenarios are available in a supplemental file (Additional file 1). The R functions and scripts used to simulate the trials and summarise the results are available at https://github.com/jatotterdell/OPTIMUMsims.

**Fig. 2:**
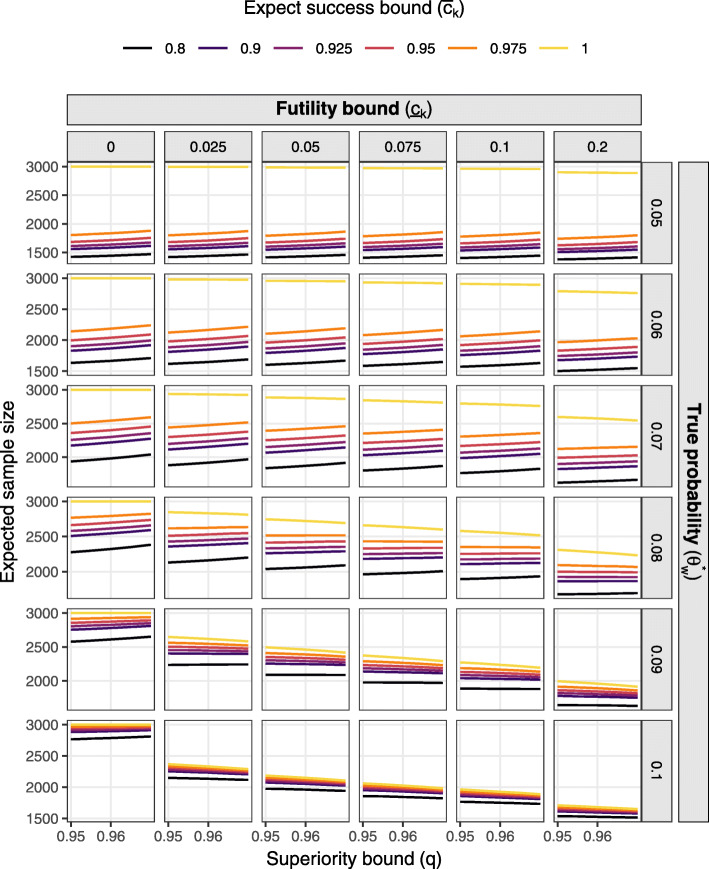
Expected sample size by decision thresholds and effect size assuming constant 16 per week accrual

**Fig. 3:**
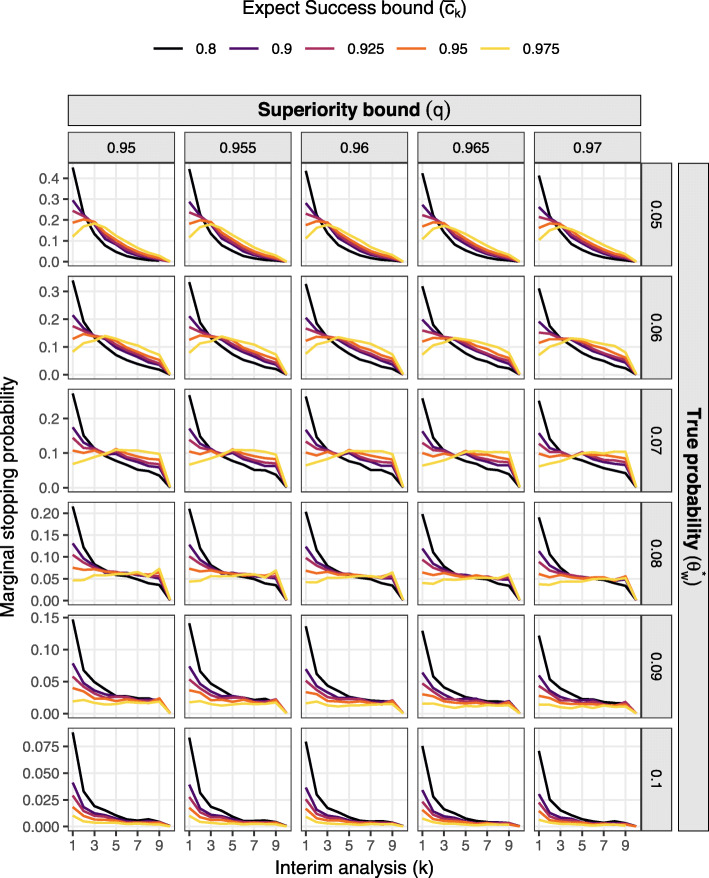
Marginal stopping probability for expected success by stage, effect size, and thresholds assuming constant 16 per week accrual and futility bound $\underline {c}=0.05$

**Fig. 4:**
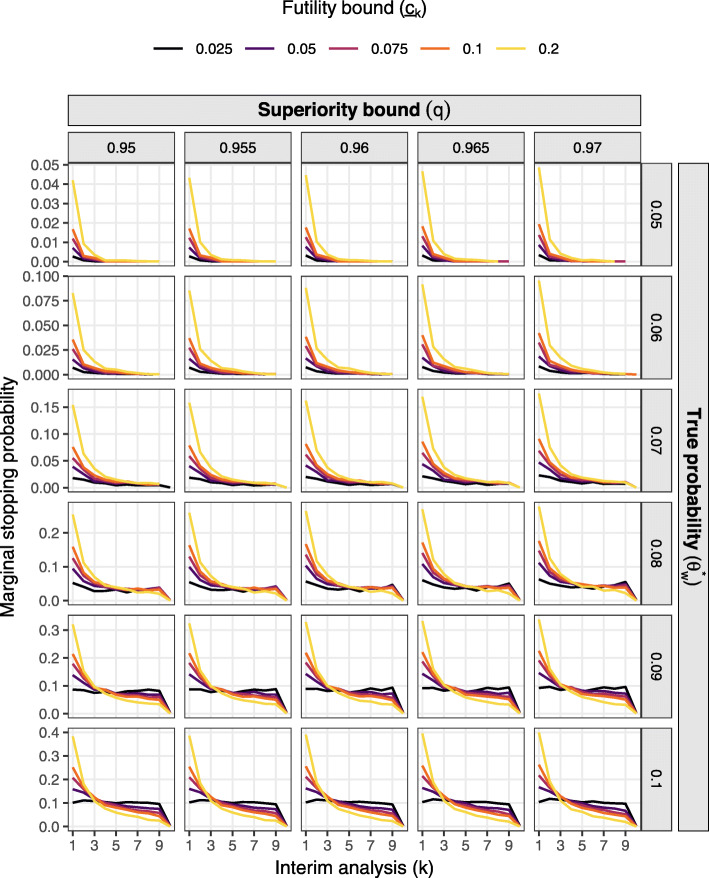
Marginal stopping probability for futility by stage, effect size, and thresholds assuming constant 16 per week accrual and expected success bound $\overline {c}=0.95$

Figures 1 and 2 present the probability of deciding superiority at the final analysis (power) and expected sample size respectively over the grid of effect sizes and decision thresholds assuming constant accrual of 16 participants per week. Figures 3 and 4 present marginal stopping probabilities for expected success and futility respectively.

Table 3 summarises the operating characteristics assuming thresholds $q=0.95, \underline {c}=0.05$, and $\overline {c}=0.95$ which were the thresholds chosen for the trial. False positives occurred with approximate probability of 0.05. The estimated probability of correctly declaring success was 0.85 when the effect was a reduction (by 30%) from 0.1 to 0.07 in the primary outcome probabilities.

**Table 3:** Trial operating characteristics assuming constant accrual of 16 per week, where $q=0.95, \underline {c}=0.05$, and $\overline {c}=0.95$

$\theta _{a}^{\star }$	$\theta _{w}^{\star }$	Decide superior	Stop early superior	No stop superior	Stop futile	Stop expect success	Superior following futile	Superior following expect success	Expected sample size
0.10	0.05	0.99	0.97	0.02	0.01	0.97	0.61	1.00	1673
	0.06	0.97	0.84	0.12	0.03	0.84	0.34	0.99	1959
	0.07	0.85	0.57	0.27	0.08	0.59	0.14	0.95	2251
	0.08	0.55	0.28	0.28	0.21	0.31	0.05	0.86	2412
	0.09	0.22	0.09	0.12	0.44	0.13	0.01	0.68	2353
	0.10	0.05	0.02	0.03	0.69	0.04	0.00	0.47	2129

## Discussion

The motivations, context, and procedures for implementing the OPTIMUM trial have been described in the protocol [2]. In this document, we have provided a detailed specification of the statistical analysis plan relating to the interim and final analyses. We presented the results of Monte Carlo simulations used to explore trial operating characteristics of the trial which were used in trial planning.

Publication of the study protocol and the present statistical analysis plan before knowledge of any results was done to enhance transparency.

## Supplementary Information

**Additional file 1** Operating characteristics for other accrual scenarios. This file includes simulation results for the other scenarios described in the main text.

## Data Availability

The investigators will undertake to make patient-level data available for independent analysis subject to any requisite approval from the relevant ethics and governance committees.

## Abbreviations

AE: Adverse event
SAE: Serious adverse event
aP: Acellular pertussis
AR: Adverse reaction
CI: Confidence interval
CrI: Credible interval
DTaP: Diphtheria tetanus acellular pertussis
DTwP: Diphtheria tetanus whole-cell pertussis
FHA: Filamentous haemagglutinin
GMC: Geometric mean concentration
GMR: Geometric mean ratio
HiB: *Haemophilus influenzae* type b
IA: Interim analysis/analyses
IQR: Inter-quartile range
ITT: Intention-to-treat
OFC: Oral food challenge
OR: Odds ratio
PP: Per-protocol
PRN: Pertactin
PRP: Polyribosyl-ribitol phosphate
PT: Pertussis toxin
SAP: Statistical analysis plan
SPT: Skin prick test
TT: Tetanus toxoid
wP: Whole-cell pertussis
