# Supplementary material for: Statistical analysis plan for the OPTIMUM study: optimising immunisation using mixed schedules, an adaptive randomised controlled trial of a mixed whole-cell/acellular pertussis vaccine schedule
Source: Trials. 2022 Feb 7;23:121. doi: 10.1186/s13063-021-05874-6 (PMC8819850; doi:10.1186/s13063-021-05874-6)

Additional file 1 — Operating characteristcs for other accrual scenarios

1 Constant accrual of 5 participants per week

This section presents the trial operating characteristics assuming a slower than anticpated accrual of 5 participants per week.

Table 1: Trial operating characteristics assuming constant accrual of 5 per week, where $q = 0.95$, $\underline{c} = 0.05$, and $\overline{c} = 0.95$.

$\theta_a^\star$	$\theta_w^\star$	Decide superior	Stop early superior	No stop superior	Stop futile	Stop expect success	Superior following futile	Superior following exepct success	Expected sample size
0.10	0.05	0.98	0.97	0.00	0.01	0.99	0.07	0.99	1014
	0.06	0.95	0.93	0.02	0.02	0.95	0.02	0.98	1305
	0.07	0.83	0.73	0.10	0.10	0.76	0.00	0.96	1676
	0.08	0.56	0.44	0.13	0.29	0.46	0.00	0.94	1933
	0.09	0.24	0.16	0.08	0.59	0.19	0.00	0.86	1865
	0.10	0.07	0.05	0.02	0.84	0.06	0.00	0.81	1574

Figure 1: Probability of deciding superiority at the final analysis by decision thresholds and effect size assuming constant 5 per week accrual.

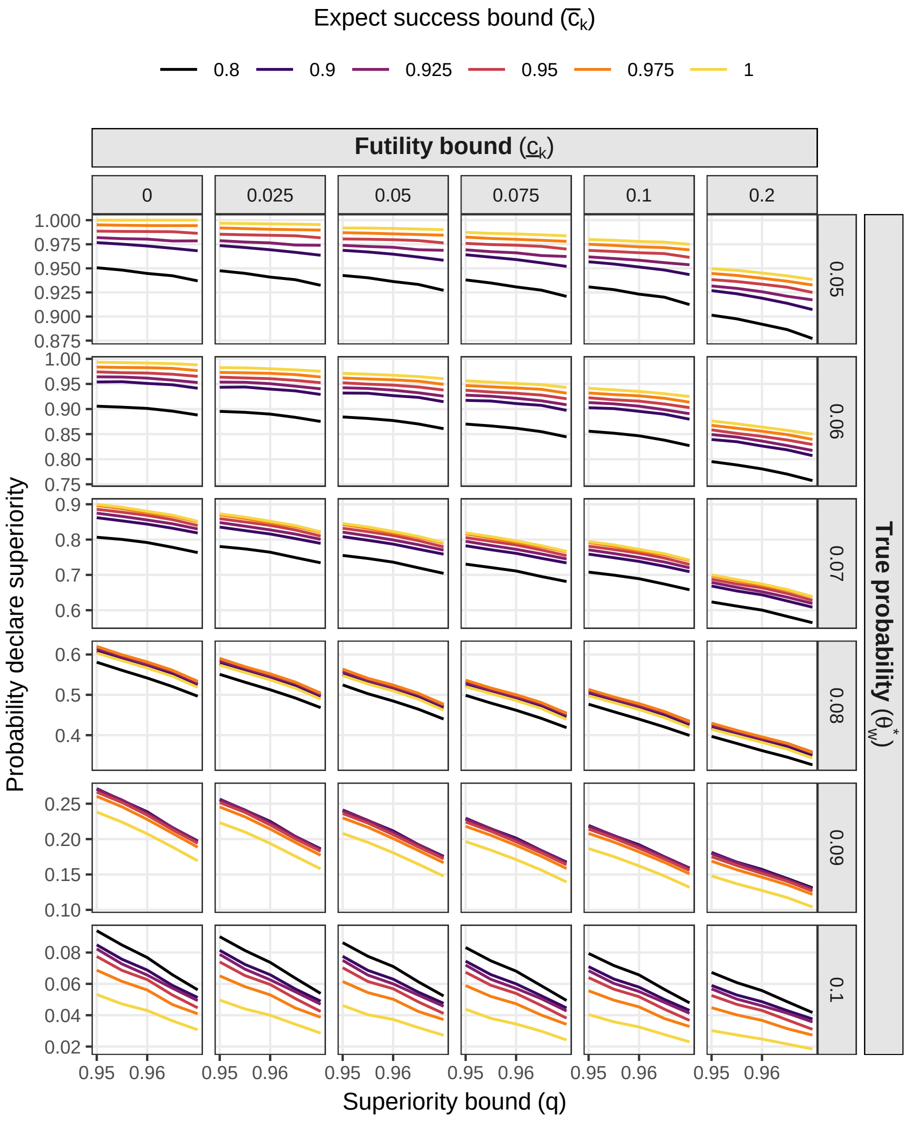

Figure 2: Expected sample size by decision thresholds and effect size assuming constant 5 per week accrual.

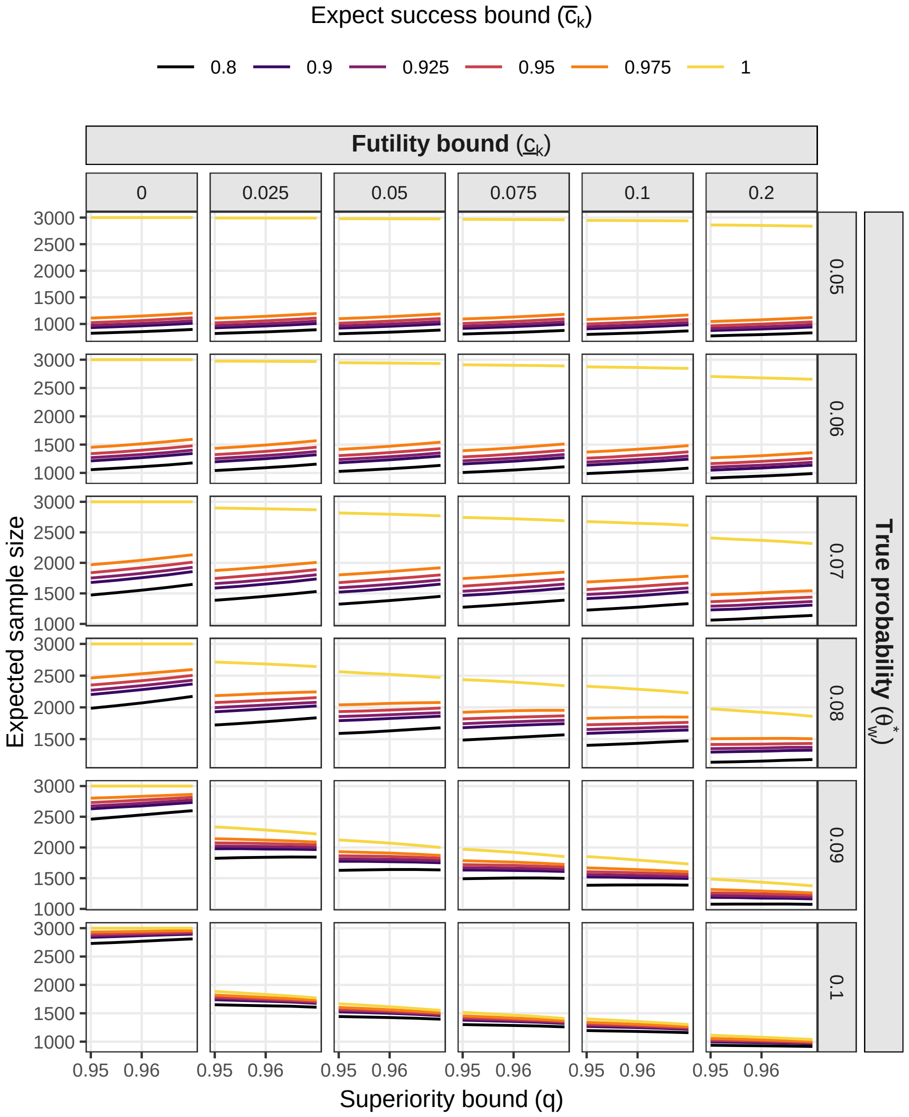

Figure 3: Marginal stopping probability for expected success by stage, effect size, and thresholds assuming constant 5 per week accrual and futility bound $\underline{c} = 0.05$.

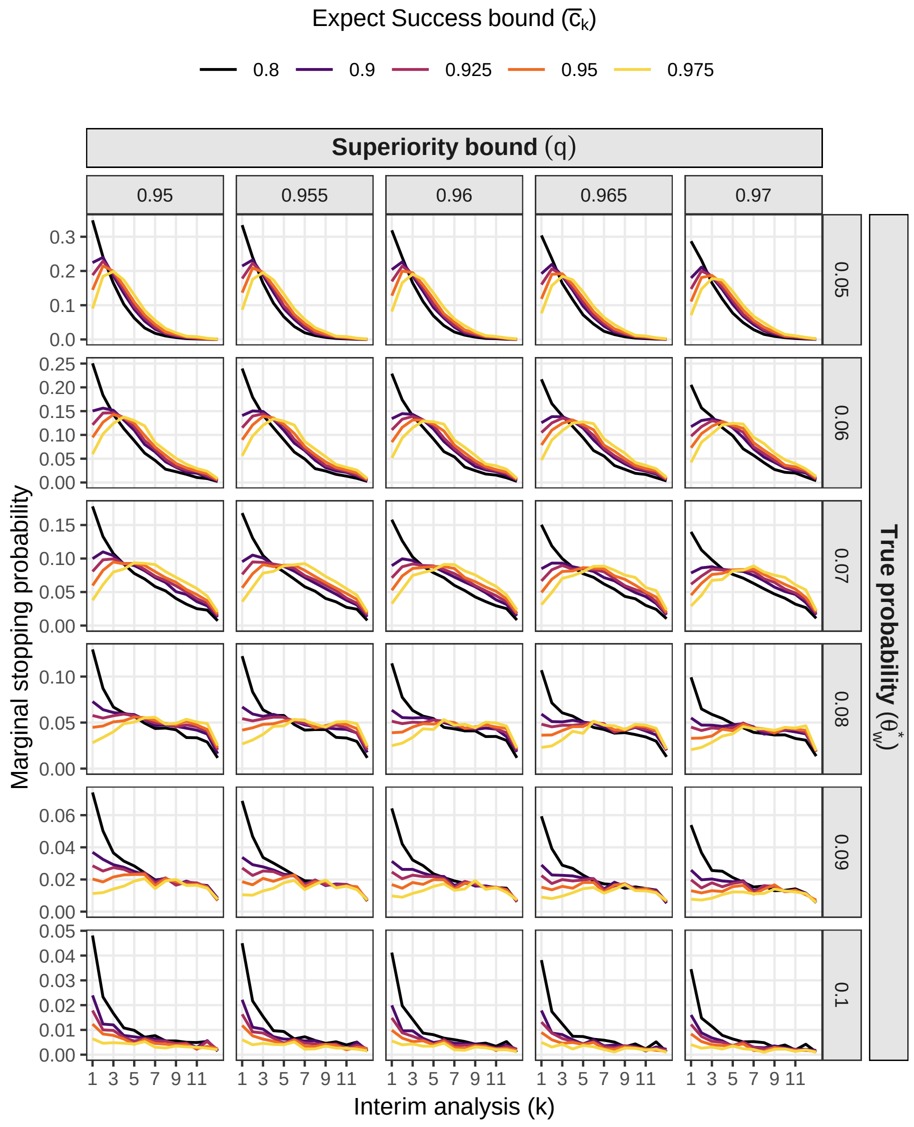

Figure 4: Marginal stopping probability for futility by stage, effect size, and thresholds assuming constant 5 per week accrual and expected success bound $\bar{c} = 0.95$.

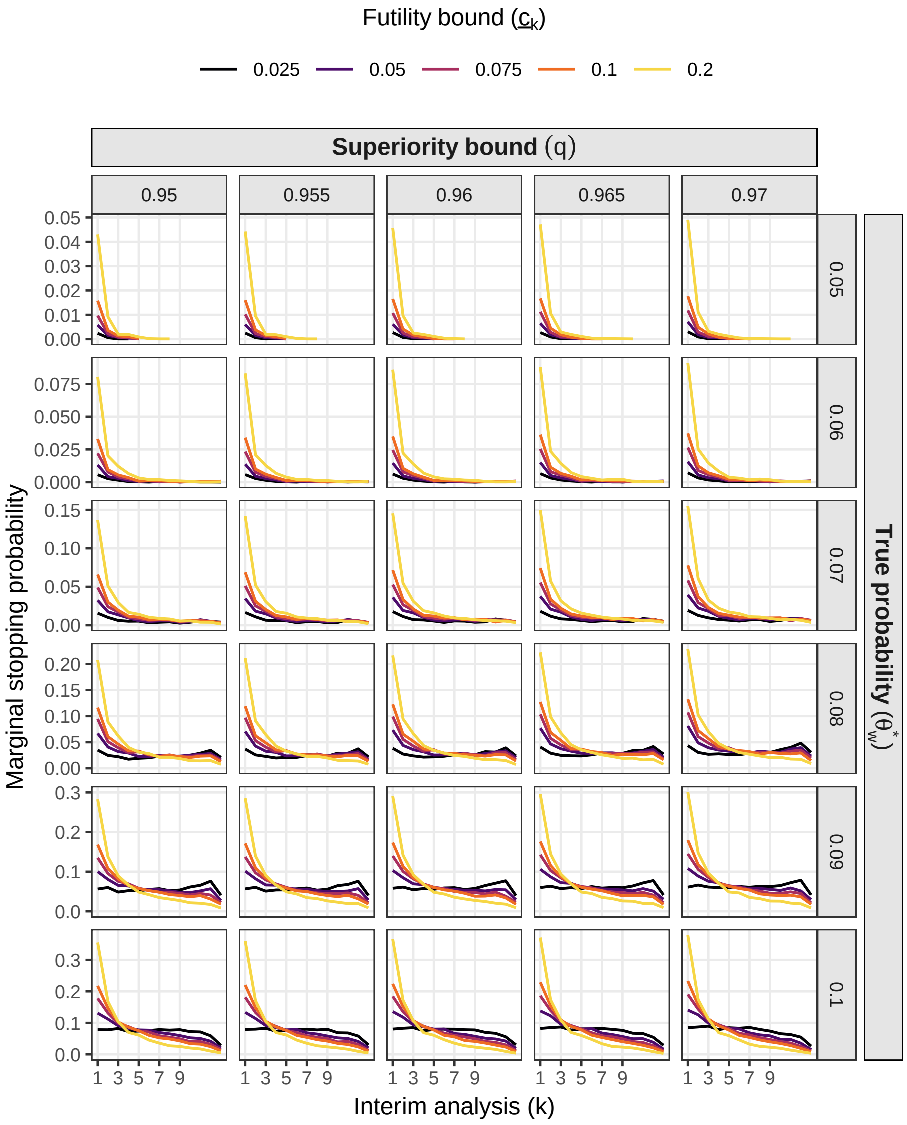

2 Ramp-up of accrual over time

This section presents the trial operating characteristics assuming a ramp-up in accrual over time. Initially, it is assumed that accrual is slow over the first 12 months of the study before gradually increasing to a fixed accrual rate after 3 years.

Table 2: Trial operating characteristics assuming a ramp-up of accrual over time, where $q = 0.95$, $\underline{c} = 0.05$, and $\overline{c} = 0.95$.

$\theta_a^\star$	$\theta_w^\star$	Decide superior	Stop early superior	No stop superior	Stop futile	Stop expect success	Superior following futile	Superior following exepct success	Expected sample size
0.10	0.05	1.00	0.91	0.09	0.01	0.90	0.76	1.00	1985
	0.06	0.97	0.72	0.25	0.02	0.72	0.52	0.99	2267
	0.07	0.86	0.45	0.41	0.07	0.46	0.25	0.95	2479
	0.08	0.56	0.21	0.35	0.17	0.24	0.09	0.83	2586
	0.09	0.23	0.07	0.16	0.35	0.10	0.02	0.63	2534
	0.10	0.05	0.02	0.03	0.57	0.04	0.01	0.36	2361

Figure 5: Probability of deciding superiority at the final analysis by decision thresholds and effect size assuming ramp-up accrual.

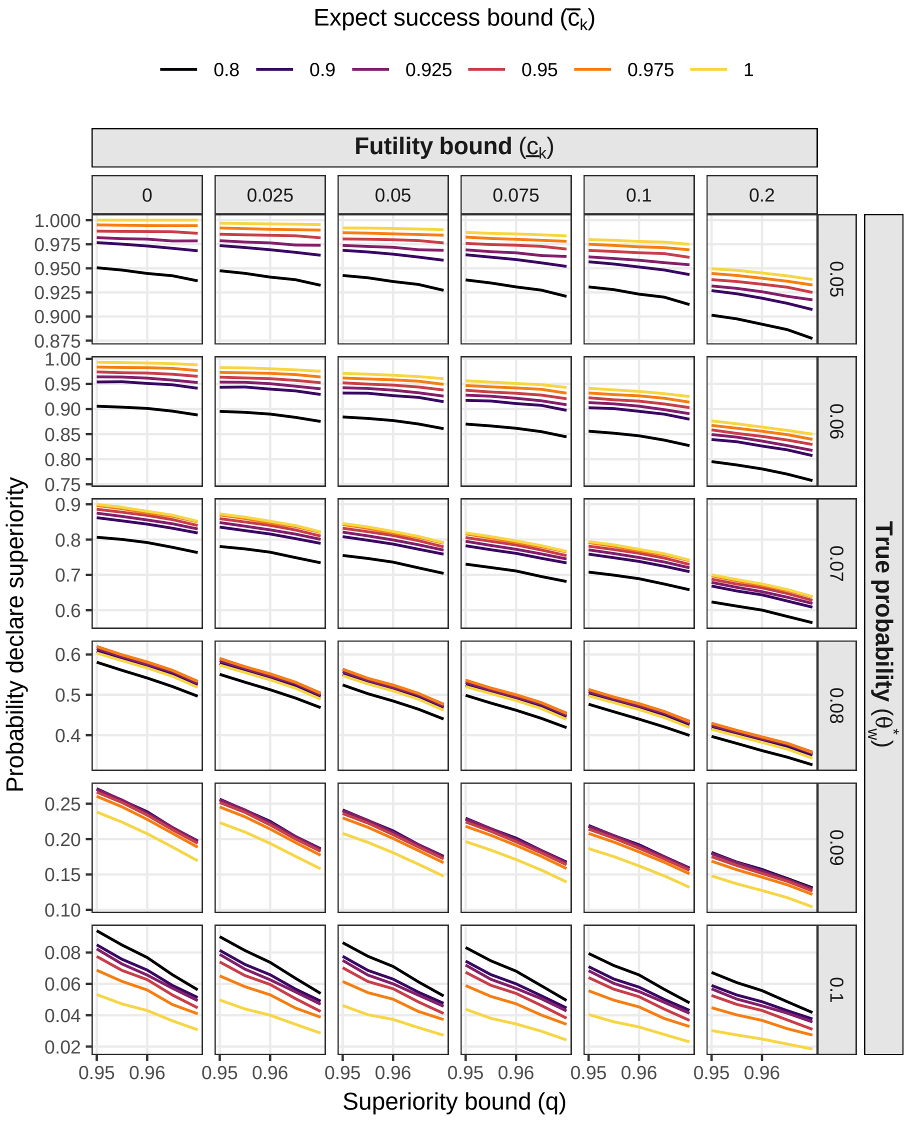

Figure 6: Expected sample size by decision thresholds and effect size assuming ramp-up accrual.

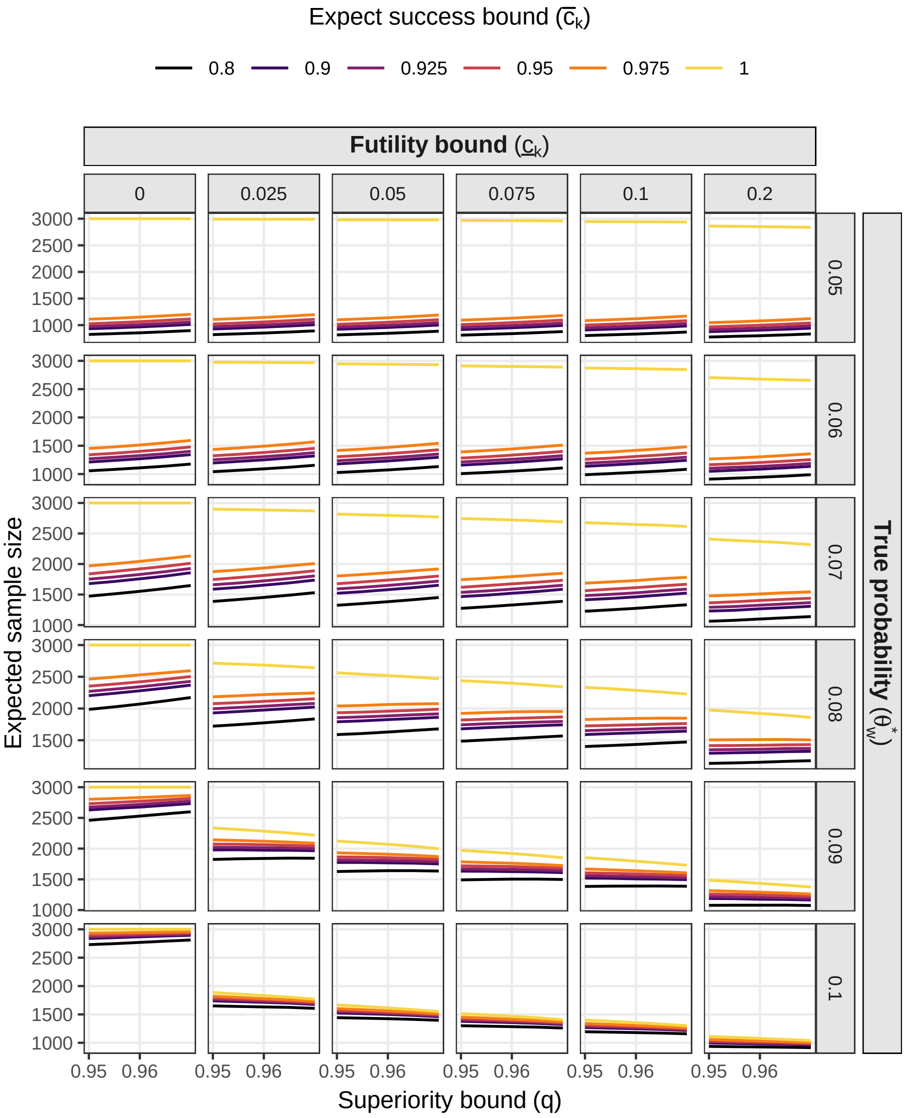

Figure 7: Marginal stopping probability for expected success by stage, effect size, and thresholds assuming ramp-up accrual and futility bound $\underline{c} = 0.05$.

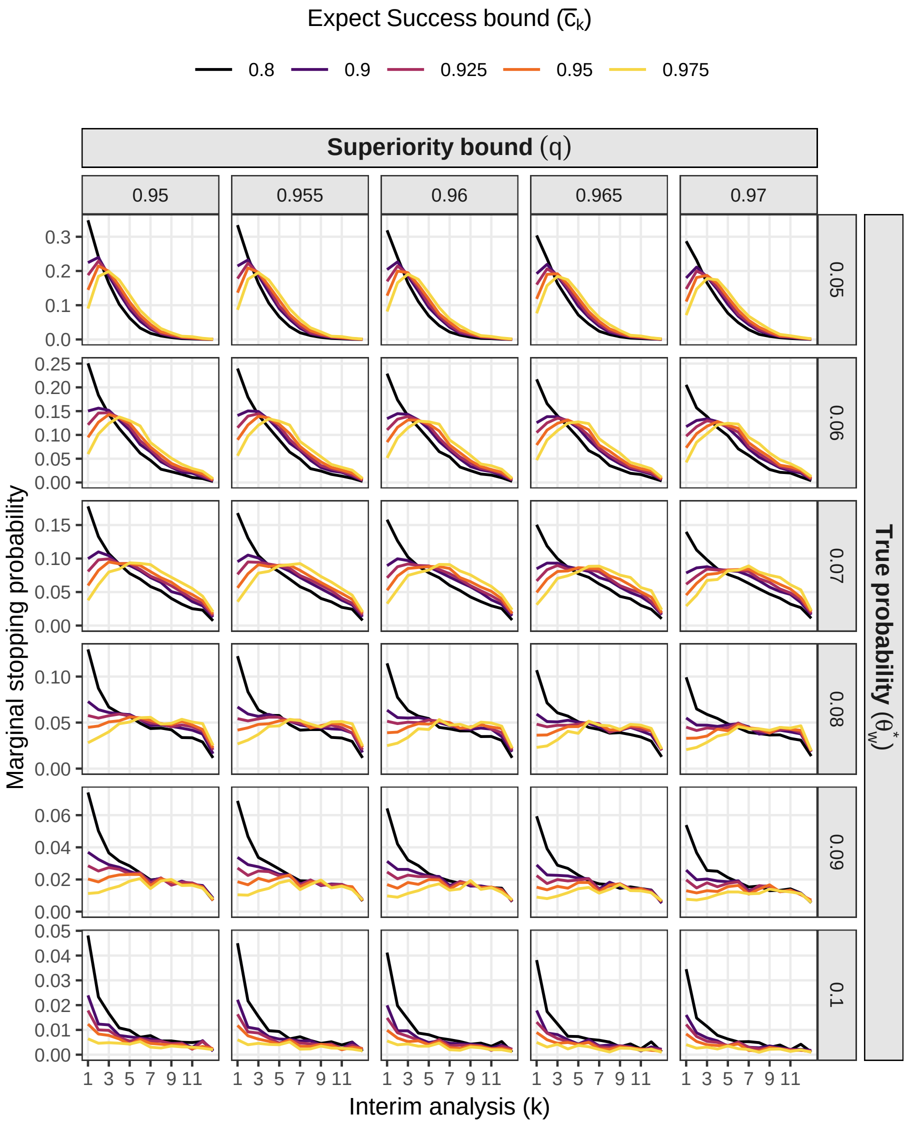

Figure 8: Marginal stopping probability for futility by stage, effect size, and thresholds assuming ramp-up accrual and expected success bound $\bar{c} = 0.95$.

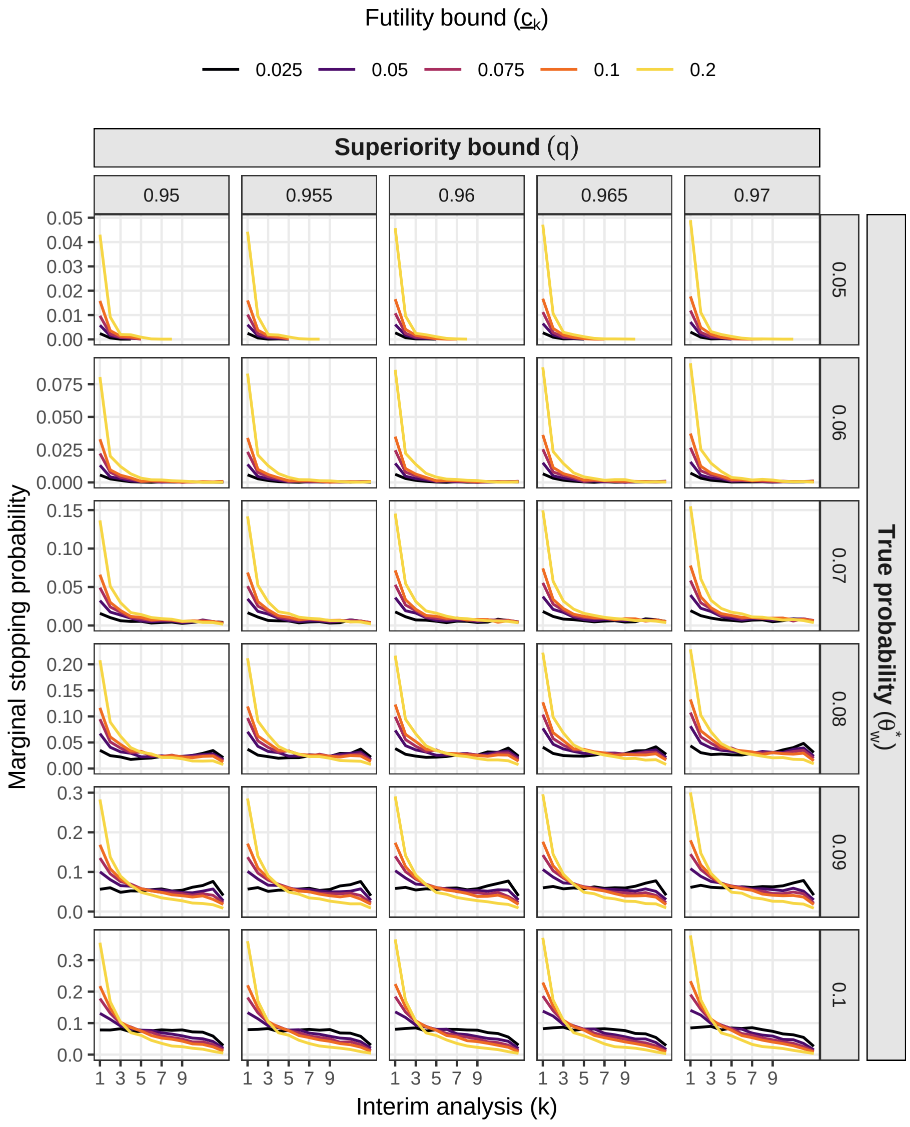

Supplement: Supplementary file 1 — Additional file 1 Operating characteristics for other accrual scenarios. This file includes simulation results for the other scenarios described in the main text. [file 13063_2021_5874_MOESM1_ESM.pdf]
